# Acoustic rhinometry after nasal provocation test, 6 months interim analysis of alumites study, a randomized, controlled, multicentre phase IV study with house dust mites subcutaneous immunotherapy

**DOI:** 10.1186/2045-7022-3-S2-P43

**Published:** 2013-07-16

**Authors:** Jaime Sanchez, Carmen Vidal, Dolores Hernandez, Antonio Valero, Jose Vicente Castello, Antonio Pelaez, Mario Alberto Garcia

**Affiliations:** 1 Hospital Clinic Universitari de Barcelona, Allergy Service, Barcelona, Spain; 2 Complejo Hospitalario de Santiago. Hospital del Conxo, Allergy Service, Santiago de Compostela, Spain; 3 Hospital Universitario y Politecnico La Fe, Allergy Service, Valencia, Spain; 4 Hospital General de Castellon, Allergy Service, Castellon, Spain; 5 Hospital Clinico Universitario de Valencia, Allergy Service, Valencia, Spain; 6 Stallergenes Iberica, Medical Department, Barcelona, Spain

## Background

ALUMITES study was designed to assess the efficacy of house dust mites (HDM: *D. pteronyssinus* + *D. farinae*) subcutaneous immunotherapy for the treatment of allergic rhinitis patients along one year. Here we report an interim analysis done after 6 months of treatment.

## Methods

In this controlled multicentre phase IV study, HDM adult allergic patients were randomized to receive subcutaneous immunotherapy with a 10 IR/ml depot extract plus symptomatic treatment (group A) or only symptomatic treatment (group B) (2:1). Acoustic rhinometry after nasal provocation test (NPT) was selected to objectively assess the efficacy of this treatment by measuring the nasal volume (NV) and minimum cross-sectional area (MCA). A visual analogic scale (VAS) has been used to subjectively assess the efficacy (0-10 scale, lower is better)NPT consists in 5 determinations at intervals of 15 minutes (basal, diluent, 0.01 IR, 0.1 IR, 1 IR) and one last determination 30 minutes after the last administration. The extract used for the NPT was 100 IR/ml of *D. pteronyssinus*.

## Results

Data from 38 of 57 patients were complete to be analyzed. MCA difference from baseline to six months visit was 1.6 cm^2^ (95% CI: [-2.1, 5.3]) in active group and 0.8 cm^2^ (95% CI: [-4.3, 5.9]) in control group. NV difference from baseline to six months visit was 37.1 cm_3_ (95% CI: [-16.9, 91.2]) in active group and -5.8 cm3 (95% CI: [-75.6, 64]) in control group. The analysis of covariance (ANCOVA) showed statistical differences in basal determination of MCA after 6 months treatment (p=0.0001).The symptoms score after the last provocation of 1 IR measured by VAS from baseline to the six month visit had a reduction of -2.4 points (95% CI: [-3.2, -1.6], p<0.0001) in the active group whereas there was no statistical reduction in the control group: -0.7 points (95% CI: [-2.5, 1.0], p=0.577).

## Conclusion

Despite of the final low sample to analyze, efficacy is demonstrated with six months of SCIT treatment with 10 IR/ml depot HDM extract that resulted in a significant improve in clinical symptoms, this improvement was not achieved by the control group. Objective assessment of efficacy shows a clear positive trend in minimal cross-sectional area determination.

